# Complete Genome Sequences of Two Porcine Deltacoronavirus Strains, CHN-GD16-03 and CHN-GD16-05, Isolated in Southern China, 2016

**DOI:** 10.1128/genomeA.01545-17

**Published:** 2018-01-25

**Authors:** Kaijie Mai, Di Li, Jiaoling Wu, Zixian Wu, Jian Cheng, Lili He, Xiaoyu Tang, Zhihai Zhou, Yuan Sun, Jingyun Ma

**Affiliations:** a Key Laboratory of Animal Health Aquaculture and Environmental Control, College of Animal Science, South China Agricultural University, Guangzhou, Guangdong, People’s Republic of China; b Guangdong Wen’s Foodstuff Group Co., Ltd., Xinxing, Guangdong, People’s Republic of China

## Abstract

We report here the amplification and sequence analysis of two complete genomes of newly emerged porcine deltacoronavirus (PDCoV) strains, isolated from diarrhea samples from piglets in Guangdong Province in southern China. These genomes provide further sequence data for evaluating the relationships among PDCoVs from different countries.

## GENOME ANNOUNCEMENT

Porcine deltacoronavirus (PDCoV) is an enveloped positive-sense single-stranded RNA virus that causes enteric, respiratory, and other diseases, such as porcine epidemic diarrhea virus (PEDV) and transmissible gastroenteritis virus (TGEV), *in vivo* in pigs. It was first detected in Hong Kong, China (1), and emerged later in the United States (2–4). It has now spread to South Korea, mainland China, and several countries of Southeast Asia (5–9), causing economic losses to the global commercial swine industry.

From December 2015 to June 2016, an outbreak of diarrhea in piglets was discovered among 11 commercial swine farms in Guangdong, southern China. During our epidemiological investigation of the outbreak (10), 252 fecal and intestinal samples were collected from sucking piglets (*n* = 203) and sows (*n* = 49) from these 11 swine farms and screened for PDCoV, PEDV, TGEV, and rotavirus A. We designed 16 pairs of primers on the basis of PDCoV strain Illinois121 from the United States (GenBank accession no. KJ481931) to expand the full-length genome of two PDCoV strains that were isolated and purified twice and defined as CHN-GD16-03 and CHN-GD16-05. Multiple sequence alignment and phylogenetic analysis of the complete nucleotide sequences of the two strains were performed and constructed with MEGA 7.

The genomes of PDCoV strains CHN-GD16-03 and CHN-GD16-05 are 25,396 bp and 25,403 bp in length, respectively, excluding the 3′ poly(A) tails, and are arranged as follows: 5′ untranslated region (UTR), open reading frame 1ab, spike (S), envelope (E), membrane (M), nonstructural protein 6 (NS6), nucleocapsid (N), NS7, and 3′ UTR. A single A deletion presented in the 5′ UTR of strain CHN-GD16-05, which was also found in strains S5011 and S5015L from Thailand and strain P1_16_BTL_0115 from Laos, while a unique 8-nucleotide (nt) deletion (AATCTATG) was found in the 3′ UTR of strain CHN-GD16-03. Analysis of the full-length sequences revealed that strain CHN-GD16-03 had the highest nt identity (99.1%) with strain CHN-HB-2014 from China, while strain CHN-GD16-05, with an nt identity of 99.22%, was most closely related to strain KY4813 from the United States. Phylogenetic analysis results showed that strain CHN-GD16-05 was located in the U.S. branch. Interestingly, strain CHN-GD16-03 belonged to the Chinese group at the complete genome level, but, based on the S gene, it was proved to belong to the Thai and Laotian branch. The complete S gene of this strain shared 97.0% identity with that of strain P1_16_BTL_0115 (Laos), but the remainder of the genome shared 98.8% to 99.5% nt identities with CHN-JS-2014 (China). After analysis with RDP4, recombinant breakpoints were detected mapping from position 19251 to 23074, in which the coding regions of the S and E genes were located.

In conclusion, phylogenetic analysis suggested that PDCoV strain CHN-GD16-05 had a close relationship with U.S. and South Korean strains, and, importantly, the newly discovered suspected recombinant strain CHN-GD16-03 complements the geographical lineage theory of PDCoV distribution in the world.

### Accession number(s).

The complete genome sequences of the isolates CHN-GD16-03 and CHN-GD16-05 have been deposited in GenBank under the accession no. KY363867 and KY363868, respectively.
